# Differential neural reward reactivity in response to food advertising medium in children

**DOI:** 10.3389/fnins.2023.1052384

**Published:** 2023-02-01

**Authors:** Dabin Yeum, Courtney A. Jimenez, Jennifer A. Emond, Meghan L. Meyer, Reina K. Lansigan, Delaina D. Carlson, Grace A. Ballarino, Diane Gilbert-Diamond, Travis D. Masterson

**Affiliations:** ^1^Department of Biomedical Data Science, Geisel School of Medicine at Dartmouth College, Lebanon, NH, United States; ^2^Department of Epidemiology, Geisel School of Medicine at Dartmouth College, Lebanon, NH, United States; ^3^Department of Psychological and Brain Science at Dartmouth College, Hanover, NH, United States; ^4^Department of Pediatrics, Geisel School of Medicine at Dartmouth College, Lebanon, NH, United States; ^5^Department of Psychology, Columbia University, New York, NY, United States; ^6^Department of Medicine, Geisel School of Medicine at Dartmouth College, Lebanon, NH, United States; ^7^Department of Nutritional Sciences, College of Health and Human Development, The Pennsylvania State University, University Park, PA, United States

**Keywords:** food cues, fMRI, neural reactivity, visual stimuli, children, static ad, dynamic ad

## Abstract

**Introduction:**

Food cues including food advertisements (ads) activate brain regions related to motivation and reward. These responses are known to correlate with eating behaviors and future weight gain. The objective of this study was to compare brain responses to food ads by different types of ad mediums, dynamic (video) and static (images), to better understand how medium type impacts food cue response.

**Methods:**

Children aged 9–12 years old were recruited to complete a functional magnetic resonance imaging (fMRI) paradigm that included both food and non-food dynamic and static ads. Anatomical and functional images were preprocessed using the fMRIPrep pipeline. A whole-brain analysis and a targeted region-of-interest (ROI) analysis for reward regions (nucleus accumbens, orbitofrontal cortex, amygdala, insula, hypothalamus, ventral tegmental area, substantia nigra) were conducted. Individual neural responses to dynamic and static conditions were compared using a paired *t*-test. Linear mixed-effects models were then constructed to test the differential response by ad condition after controlling for age, sex, BMI-z, physical activity, and % of kcal consumed of a participant’s estimated energy expenditure in the pre-load prior to the MRI scan.

**Results:**

A total of 115 children (mean=10.9 years) completed the fMRI paradigm. From the ROI analyses, the right and left hemispheres of the amygdala and insula, and the right hemisphere of the substantia nigra showed significantly higher responses for the dynamic food ad medium after controlling for covariates and a false discovery rate correction. From the whole-brain analysis, 21 clusters showed significant differential responses between food ad medium including the precuneus, middle temporal gyrus, superior temporal gyrus, and inferior frontal gyrus, and all regions remained significant after controlling for covariates.

**Discussion:**

Advertising medium has unique effects on neural response to food cues. Further research is needed to understand how this differential activation by ad medium ultimately affects eating behaviors and weight outcomes.

## Introduction

In the United States, the prevalence of obesity was estimated to be 19.7% among children and adolescents aged 2–19 years between 2017 and 2020 according to the National Health Statistics Report (Bryan et al., 2021). Obesity in childhood often continues into adulthood (Singh et al., 2008), and increased body weight is related to chronic disease including type 2 diabetes, cardiovascular disease, and hypertension (National Institutes of Health, 1998; Dietz, 2004; Daniels et al., 2005; Freedman et al., 2007). Many studies have indicated that food marketing plays a critical role in promoting the current obesity epidemic particularly in amplifying the obesogenic environment that younger children in the United States find themselves in Harris et al. (2009), Zimmerman (2011), Ustjanauskas et al. (2013), Harris and Kalnova (2018), Coates et al. (2019), Bragg et al. (2021), and Packer et al. (2022).

For children specifically, media is the primary source of food marketing (World Health Organization Regional Office for Europe, 2016; Anderson, 2018; Edwards et al., 2022). In 2021, children aged 8–12-years spent 4–6 h a day watching entertainment and using apps that include food marketing materials including smartphones, tablets, gaming consoles, TVs, and computers (The American Academy of Child and Adolescent Psychiatry [AACAP], 2020; Rideout et al., 2022). Specifically, between 2019 and 2021 the total amount of time spent on screen media in US children marked a much faster increase than the previous 4 years with the biggest increases seen in screen time activities including watching online videos, using social media, and browsing websites (Rideout et al., 2022). Through media, children are exposed to environmental food cues in the form of food advertisements, frequently of unhealthy foods and beverages (Elsey and Harris, 2016; Frazier and Harris, 2018). According to national Nielsen data, the average U.S. child or adolescent had viewed over 4,300 TV food ads on TV platforms in 2017 with an average of 10 food-related TV ads per day (Frazier and Harris, 2018). Children and adolescents are now constantly exposed to advertisements through digital media on smartphones, tablets, and laptops (World Health Organization Regional Office for Europe, 2016). Online marketing is presented in a decidedly unique way with a combination of both static and dynamic advertising being presented on popular entertainment,^1^ social media,^2^ and streaming^3^ websites (Kelly et al., 2008; Guo et al., 2019). Static advertising is often found in the form of banner, sidebar, and click-through ads, which utilize eye-catching visuals and catch-phrases to increase brand exposure and familiarity that might impact viewers’ food choice and intake (Pieters and Wedel, 2004; Edquist et al., 2011). The various layers integrated in this complex marketing environment make it especially challenging to investigate the impact of unhealthy food advertising exposure on younger children (Elsey and Harris, 2016; Tatlow-Golden et al., 2021).

Images in both dynamic and static ads serve as general food cues. Food cues, including those in food marketing, are known to generally activate the dopaminergic mesolimbic pathway of the brain which has implications to health (Stoeckel et al., 2008; Castellanos et al., 2009; Demos et al., 2012; Lawrence et al., 2012; Wagner et al., 2012; Gearhardt et al., 2014; Rapuano et al., 2016, 2017). For instance, regions of the mesolimbic pathway have been previously associated with reward based outcomes (Bassareo et al., 2015) and therefore may influence food intake or other food related behaviors which are tied to reward pathways (Douglass et al., 2017; Liu and Kanoski, 2018; Bond et al., 2020). According to previous functional magnetic resonance imaging (fMRI) research, brain response to food stimuli is also associated with memory, cognitive evaluation of salient stimuli, and overall decision making (Camara, 2008; Schultz, 2015). Previously implicated regions from these studies include nucleus accumbens, orbitofrontal cortex, amygdala, insula, hypothalamus, substantia nigra, and ventral tegmental area (Killgore et al., 2003; Kelley et al., 2005; Berridge, 2009; Kenny, 2011; van der Laan et al., 2011; García-García et al., 2013; Huerta et al., 2014; Meye and Adan, 2014; Sheng et al., 2014; Masterson et al., 2016; van Meer et al., 2016). These regions are also known to be related to food craving and appetitive motivation (Pelchat et al., 2004; Siep et al., 2012; Dietrich et al., 2016; Kahathuduwa et al., 2018; Contreras-Rodríguez et al., 2019). Moreover, activity in these dopaminergic reward regions in response to food cues has been previously associated with increased food consumption (Lawrence et al., 2012; Masterson et al., 2019b; Gearhardt et al., 2020) and weight gain (Stice et al., 2010; Demos et al., 2012; Gearhardt et al., 2014).

Thus far, previous studies focused on comparison of food cues such as food logos vs. non-food logos (Bruce et al., 2013, 2014; Fehse et al., 2017; Masterson et al., 2019b) and food commercials vs. non-food commercials (Gearhardt et al., 2014, 2020; Bruce et al., 2016; Rapuano et al., 2016, 2017; Masterson et al., 2019a); however, no studies have compared the differential reward activation between static and dynamic advertising mediums. While static advertisements present powerful graphics and eye-catching texts that elicit reward responses (Pieters and Wedel, 2004; Edquist et al., 2011), dynamic advertisements also include narrative and emotional aspects and soundtracks, that may heighten the reward response (Emond et al., 2015). Understanding if one type of medium is more effective at producing a neural response indicative of reward reactivity is critical to inform policies related to child-directed food marketing online.

Therefore, we sought to compare brain responses between food-related dynamic and static advertisements using a fMRI paradigm. We hypothesized that dynamic advertisements would elicit greater food-specific neural response in the reward regions than static advertisements. We conducted a targeted region of interest (ROI) analysis using *a priori* ROIs identified from previous literature as we hypothesized reward regions of the brain would be highly relevant to food cues. Additionally, we also conducted a non-hypothesis-driven whole-brain exploratory analysis to consider differences that may be related to medium type, independent of content.

## Materials and methods

### Study participants

**FIGURE 1:**
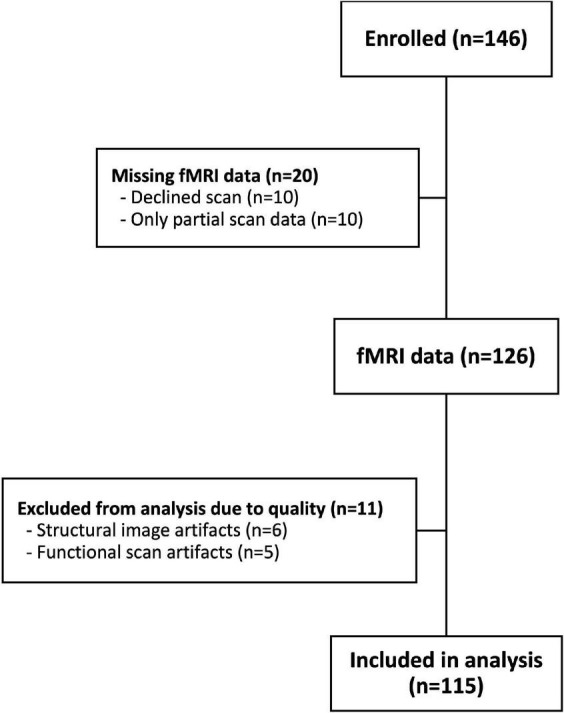
Participant flow chart.

This study utilized data from a larger study with the primary goal to investigate the relationship between children’s response to dynamic food ads and snack food intake. A total of 146 children between the ages of 9 and 12 were enrolled. Children were recruited through community fliers, listservs, and events. Participants were excluded from the present study if they had allergies or dietary restrictions related to the foods served in the study meals, metal in or on the body, claustrophobia, psychiatric or neurological disorders, had appetite-or attention-altering disorders, or if they were not fluent in English. Additionally, participants were excluded if they had an immediate family member who had previously participated in the study. Caregivers and children provided informed consent and assent, and participants received monetary compensation for participating. Dartmouth’s Committee for the Protection of Human Subjects approved all study protocols. A participant flow chart is shown in Figure 1. Data from 31 children were excluded due to: refusal to be scanned (*n* = 10); incomplete scans (*n* = 10); excessive movement in the scanner (*n* = 6); and functional scan artifacts (*n* = 5). Therefore, the final analysis sample consisted of 115 participants (66 male; mean age (SD) = 10.9 (1.17) years).

### Study overview

Each child, accompanied by a parent, was scheduled for a lunchtime (11:00 a.m. to 1:00 p.m.) or dinnertime (4:00 p.m. to 6:00 p.m.) appointment when they completed a brief lab assessment and then the fMRI scan. During the lab assessment, children were provided with a standardized pre-load meal to ensure fullness in line with the protocol of the larger study. Hunger level was assessed prior to the scan using the Freddy Fullness scale (Keller et al., 2006), a validated scale for estimating satiety in children. A trained research staff member then measured children’s height and weight and administered a battery of questionnaires to the parent. Following the parent questionnaires, the fMRI paradigm and protocol were explained to both children and their parents prior to conducting the scanning protocol.

### Scanning paradigm

**FIGURE 2:**
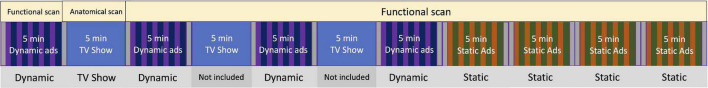
MRI paradigm. Food and non-food ads were presented which alternated in an AB/BA (randomized order) pattern during the dynamic and static runs (shown in two different colors). Each run contained five food and five non-food ads, and each ad was 15 s in length. Each fixation cross block between runs was 15 s in length. For the four dynamic runs, a total of 20 food ads (5 min) and 20 non-food ads (5 min) were presented; for the four static runs, a total of 40 food ads (5 min) and 40 non-food ads (5 min) were presented to the study participants.

The scan protocol consisted of a series of videos and images that were presented to participants using E-Prime (Psychology Software Tools Inc., Sharpsburg, PA, United States), herein called “runs.” The main portion of the protocol was designed to simulate a normal television viewing session and included four commercial runs and three 5-min TV show runs during which the children watched a popular age-appropriate science show (MythBusters). Following this naturalistic paradigm, participants viewed four runs of static ads. Participants viewed all videos and images on a screen through a mirror mounted to the head coil. Each MRI scan consisted of 12 functional runs total. For the purposes of this analysis, only eight runs (the four runs of dynamic ads and the four runs of static ads) were included in the analysis (Figure 2). Participants’ structural scans were completed during one of the TV shows runs.

Each functional run was approximately 5-min in length. Each run began and ended with a 15 s presentation of a fixation cross. To promote participant engagement, a trained research staff verbally talked with the participants between each run and asked if they would like to continue. In the dynamic ad runs, five food and five non-food TV commercials were presented which alternated in an AB pattern (Smith et al., 2007; Maus et al., 2010). The block pattern for each run was randomized (AB or BA) along with which commercials were played within each block was also randomized. Each commercial was approximately 15 s in length. Static ad runs were similarly randomized but consisted of 10 food and 10 non-food static ads. Each ad image was displayed for 7.5 s followed immediately by another image of the same type (food or non-food) which was also displayed for 7.5 s for a total exposure time of 15 s. This back-to-back display of ads was arranged so that the ad exposure period matched that of the dynamic ad length (15 s). An additional 15 s fixation cross block was placed in the middle of each static ad run to ensure equal amounts of exposure to all stimulus types (i.e., static, dynamic, and fixation).

### Image acquisition

Scanning was performed on a 3.0T Siemens MAGNETOM Prisma MRI scanner with a 32-channel head coil. The following parameters were used to obtain a T1-weighted magnetization-prepared rapid acquisition gradient echo (MPRAGE) structural scan for each participant: Echo time (TE) = 2.32 ms; repetition time (TR) = 2,300 ms; flip angle = 8°; matrix size = 256 × 256 mm; field of view = 240 × 240 mm; 192 slices; slice thickness = 0.9 mm; voxel size = 0.9 × 0.9 × 0.9 mm. Functional images were acquired using a T2*-weighted echo planar imaging (EPI) sequence using the following parameters: TE = 33 ms; TR = 1,250 ms; flip angle = 64°; matrix size = 96 × 96; field of view = 240 × 240 mm; 56 slices; slice thickness = 2.5 mm; voxel size = 2.5 × 2.5 × 2.5 mm. Eight functional runs (4 dynamic ad runs with 144 TRs and 4 static ad runs with 157 TRs each) were included in the analysis for each participant.

### Stimuli

For the ads presented in the dynamic condition, contemporary food, and toy commercials were selected based on relevance to the age group. In a previous study, children rated the commercials included here for interest and excitement and reported no overall difference in interest and excitement between the food and non-food commercials (Rapuano et al., 2016). The ads selected for the static condition were matched to the products and companies of the ads used in the dynamic condition and therefore expected to be similarly relevant and exciting for this age group.

### Model covariates

Participant height and weight were measured using a Seca 703 Medical Scale and Seca 264 Stadiometer (Hamburg, Germany). Body mass index (BMI) was calculated using U.S. Center for Disease Control (CDC) 2000 age- and sex-specific distributions (Centers for Disease Control and Prevention [CDC], 2002). Healthy weight was defined as <85th percentile, overweight was defined as ≥85th –<95th percentile and obese was defined as ≥95th percentile.

The estimated daily energy requirement (EER) was calculated for each child according to Institute of Medicine guidelines given the child’s sex, age, and measured height and weight (Institute of Medicine (U.S.), 2005). Per the larger study, children consumed a standardized pre-load meal that consisted of macaroni and cheese, apple sauce, corn, milk, and water; the calories of each item provided were defined to meet, in total, ∼25% of each child’s calculated EER while maintaining a standardized ratio across items. The total kcal consumed at pre-load was then divided by the child’s EER to derive the percent kcal (per EER) consumed at pre-load.

Caregivers reported child physical activity by answering, “During the past 7 days, on how many days was a child active for a total of at least 60 min per day?” for child’s physical activity, which was categorized as “No days,” “1 day,” “2–3 days,” “4–5 days,” and “6–7 days.” Child’s screen time on a typical weekday and a weekend day was separately reported by caregivers. The total screen time per week was calculated by multiplying the screen time on a weekday by five and a weekend day by two. The caregiver also reported their child’s date of birth, biological sex, race, and ethnicity.

### MRI pre-processing

Results included in this manuscript come from preprocessing performed using *fMRIPrep* 1.2.5 [(Esteban et al., 2017, 2019); RRID:SCR_016216], which is based on *Nipype* 1.1.6 [(Gorgolewski et al., 2011; Esteban et al., 2022); RRID:SCR_002502].

#### Anatomical data preprocessing

The T1-weighted (T1w) image was corrected for intensity non-uniformity (INU) using N4BiasFieldCorrection, (Tustison et al., 2010) and used as T1w-reference throughout the workflow. The T1w-reference was then skull-stripped using antsBrainExtraction.sh (ANTs 2.2.0), using Open Access Series of Imaging Studies (OASIS) as target template. Brain surfaces were reconstructed using recon-all [FreeSurfer 6.0.1, RRID:SCR_001847, (Dale et al., 1999)], and the brain mask estimated previously was refined with a custom variation of the method to reconcile ANTs-derived and FreeSurfer-derived segmentations of the cortical gray-matter of Mindboggle [RRID:SCR_002438, (Klein et al., 2017)]. Spatial normalization to the International Consortium for Brain Mapping (ICBM) 152 Non-linear Asymmetrical template version 2009c [(Fonov et al., 2009), RRID:SCR_008796] was performed through non-linear registration with antsRegistration [ANTs 2.2.0, RRID:SCR_004757, (Avants et al., 2008)], using brain-extracted versions of both T1w volume and template. Brain tissue segmentation of cerebrospinal fluid (CSF), white-matter (WM), and gray-matter (GM) was performed on the brain-extracted T1w using fast [FSL 5.0.9, RRID:SCR_002823, (Zhang et al., 2001)].

#### Functional data preprocessing

For each of the eight blood oxygenation-level dependent (BOLD) runs found per participant (across all tasks and sessions), the following preprocessing was performed. First, a reference volume and its skull-stripped version were generated using a custom methodology of *fMRIPrep*. The BOLD reference was then co-registered to the T1w reference using bbregister (FreeSurfer) which implements boundary-based registration (Greve and Fischl, 2009). Co-registration was configured with nine degrees of freedom to account for distortions remaining in the BOLD reference. Head-motion parameters with respect to the BOLD reference (transformation matrices, and six corresponding rotation and translation parameters) are estimated before any spatiotemporal filtering using mcflirt [FSL 5.0.9, (Jenkinson et al., 2002)]. BOLD runs were slice-time corrected using 3dTshift from AFNI 20160207 [(Cox and Hyde, 1997), RRID:SCR_005927]. The BOLD time-series, were resampled to surfaces on the following spaces: *fsaverage5*. The BOLD time-series (including slice-timing correction when applied) were resampled onto their original, native space by applying a single, composite transform to correct for head-motion and susceptibility distortions. These resampled BOLD time-series will be referred to as *preprocessed BOLD in original space*, or just *preprocessed BOLD*. The BOLD time-series were resampled to MNI152Nlin2009cAsym standard space, generating a *preprocessed BOLD run in MNI152Nlin2009cAsym space*. First, a reference volume and its skull-stripped version were generated using a custom methodology of *fMRIPrep*. Several confounding time-series were calculated based on the *preprocessed BOLD*: framewise displacement (FD), delta variation signal (DVARS) and three region-wise global signals. FD and DVARS are calculated for each functional run, both using their implementations in *Nipype* [following the definitions by Power et al. (2014)]. The three global signals are extracted within the CSF, the WM, and the whole-brain masks. Additionally, a set of physiological regressors were extracted to allow for component-based noise correction [*CompCor*, (Behzadi et al., 2007)]. Principal components are estimated after high-pass filtering the *preprocessed BOLD* time-series (using a discrete cosine filter with 128 s cut-off) for the two *CompCor* variants: temporal (tCompCor) and anatomical (aCompCor). Six tCompCor components are then calculated from the top 5% variable voxels within a mask covering the subcortical regions. This subcortical mask is obtained by heavily eroding the brain mask, which ensures it does not include cortical GM regions. For aCompCor, six components are calculated within the intersection of the aforementioned mask and the union of CSF and WM masks calculated in T1w space, after their projection to the native space of each functional run (using the inverse BOLD-to-T1w transformation). The head-motion estimates calculated in the correction step were also placed within the corresponding confounds file. All resamplings can be performed with *a single interpolation step* by composing all the pertinent transformations (i.e., head-motion transform matrices, susceptibility distortion correction when available, and co-registrations to anatomical and template spaces). Gridded (volumetric) resamplings were performed using antsApplyTransforms (ANTs), configured with Lanczos interpolation to minimize the smoothing effects of other kernels (Lanczos, 1964). Non-gridded (surface) resamplings were performed using mri_vol2surf (FreeSurfer).

### Statistical analysis

#### Subject-level analysis

Following pre-processing, participants’ individual fMRI data were analyzed using the NLTools Python package (Chang et al., 2018). For each participant’s subject-level analysis, a general linear model (GLM) was performed by constructing a design matrix, running hemodynamic response function (HRF) convolution, adding nuisance variables related to intercepts, linear and quadratic trends, motion covariates (24 motion parameters; six demeaned realignment parameters, their squares, their derivatives, and their squared derivatives), and motion spikes [motion spikes between successive TRs and global spikes that exceed an overall average intensity change by 2.5 standard deviations (SD) as a threshold]. Data were spatially smoothed using a 6 mm full-width at half maximum (FWHM) Gaussian kernel. Structural images and any functional runs that did not pass standard visual inspection due to excessive motion artifacts were excluded from the analysis (*n* = 11). From standard visual inspection, 12 participants (∼10%) had data from one functional run excluded from the dynamic condition; 20 participants (∼17%) had data from one functional run excluded from the static condition; three participants (∼2%) were missing one run each in both dynamic and static runs. However, they did not meet the criteria for overall exclusion from the analysis, therefore, three runs were included for the individual-level regression. Any functional run in which the number of spikes lead to greater than 25% of the total run length to be censored were excluded from further analyses (*n* = 0). Furthermore, any participants for whom the number of runs was missing or excluded in more than 15% in either ad condition were excluded from further analyses (*n* = 10). Individual-level regression coefficient (beta) maps were averaged across functional runs and created contrasting the activation between food ads and non-food ads to generate beta maps that reflected the unique effect of food ads for both the dynamic and static conditions. All subject-level maps were then moved forward to both whole-brain and targeted region of interest analyses.

#### Region of interest analyses

**FIGURE 3:**
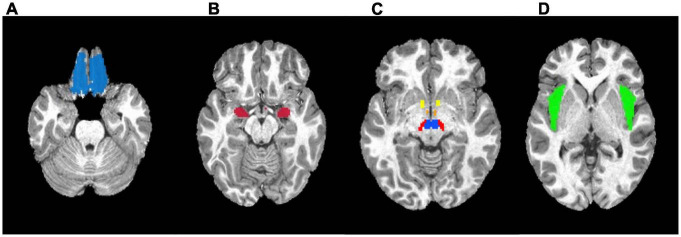
Masks used in the region-of-interest (ROI) analysis. **(A)** Orbitofrontal cortex. **(B)** Amygdala. **(C)** Yellow: Nucleus accumbens; Orange: Hypothalamus; Red: Substantia nigra; Blue: Ventral tegmental area. **(D)** Insula.

For the *a priori* region of interest (ROI) analysis, seven ROIs were selected as candidate reward regions based on previous literature (Kenny, 2011): the nucleus accumbens (NAcc), orbitofrontal cortex (OFC), amygdala, insula, hypothalamus, ventral tegmental area, and substantia nigra. Masks of these bilateral regions were generated using the Talairach Daemon and Montreal Neurological Institute (MNI) atlas using AFNI [Analysis of Functional NeuroImages version: 21.0.06 (Cox and Hyde, 1997)]. The mask of the ventral tegmental area was defined by the sphere with a radius of 5mm centered at MNI coordinate [4, −16, −10] (Carter, 2009). The ROI masks are shown in Figure 3. The beta coefficients within each ROI mask were extracted from the individual’s food > non-food beta maps from the dynamic and static conditions separately, then were averaged using 3dMaskave in AFNI. Beta values were then imported and analyzed using the R Language and Environment for Statistical Computing, version 4.0.2 (R Core Team, 2020). Using the ROI-specific beta maps, linear mixed effects (LME) models were used to test the neural response by ad condition (dynamic or static condition); a random effect was included at the participant level to account for the repeated measures within participant. Analyses were repeated, controlling for age, sex, BMI-z, physical activity, and% of kcal consumed of a participant’s estimated energy expenditure in the pre-load. LME models were fitted using the lmerTest package in R. A false discovery rate (FDR) multiple comparison correction was performed on the *p*-values in seven bilateral regions-of-interest (14 regions total) at *q* < 0.05. An additional exploratory analysis was conducted to examine the interaction between the use of binary weight status (healthy weight vs. with overweight/obesity) and the ad condition (dynamic vs. static) to explore of child weight status modified the effect of ad condition on neural response. Furthermore, a sensitivity analysis was conducted to include a total screen time per week as another covariate to account for participants’ exposure to food and non-food cues from the screen time exposure.

#### Exploratory whole-brain analysis

A whole-brain analysis was conducted using the individual beta maps as input to compare dynamic and static conditions. Initial voxel-wise paired *t*-test was conducted using individual’s food > non-food beta maps for the dynamic and static conditions with 3dttest++ in AFNI. A gray matter mask derived from the Talairach Daemon in AFNI was applied. To determine significance, an initial voxel-wise significance threshold of *p*-value < 0.001 was applied and was then cluster-corrected using a threshold of a cluster size of *k* = 180 for an overall *p*-value < 0.05. These parameters were based on 10,000 Monte-Carlo simulation determined using 3dClustSim from AFNI. The voxel with the peak response in each cluster was identified using the automated anatomical atlas (AAL) in the label4MRI package in R. Additionally, average beta coefficients were extracted from all clusters and extracted for additional analysis using LME models controlling for age, sex, BMI-z, physical activity, and% of kcal consumed of a participant’s estimated energy expenditure in the pre-load. An FDR multiple comparison correction was also applied to this set of analyses at *q* < 0.05.

## Results

A total of 115 participants were included in the analysis (Table 1). Most participants were white (93.0%) and non-Hispanic (93.9%). The average (SD) BMI Z-score was 0.60 (0.96), and 36% of participants were categorized as either having overweight or obesity.

**TABLE 1:** Baseline characteristics of study participants (*N* = 115).

	Mean (SD) or *N* (%)
Age (years)	10.9 (1.17)
**Sex**	
Female	48 (41.7%)
Male	66 (57.4%)
Prefer not to answer	1 (0.9%)
**Ethnicity**	
Hispanic	4 (3.5%)
Not Hispanic	108 (93.9%)
Prefer not to answer	2 (1.7%)
Unknown	1 (0.9%)
**Race**	
Non-white	8 (7.0%)
White	107 (93.0%)
BMI *Z*-score	0.60 (0.96)
**BMI category**	
Normal weight	74 (64.3%)
Overweight	19 (16.5%)
Obese	22 (19.1%)
**Physical activity**	
(Active for at least 60 min per day in the past 7 days)	
No days	2 (1.7%)
1 day	38 (33.0%)
2–3 days	42 (36.5%)
4–5 days	29 (25.2%)
6–7 days	1 (0.9%)
Missing	3 (2.6%)

### ROI analyses

The food > non-food contrast maps in the dynamic and static conditions were separately shown in Supplementary Figure 1. Unadjusted and adjusted LME models demonstrated differential reward activation to food ads in the dynamic condition versus static condition (Table 2). Of the 14 bilateral regions tested, six regions showed a statistically significantly higher food-related neural response in the dynamic as compared to the static condition. Specifically, in both unadjusted and adjusted models and after the FDR correction, the right and left amygdala, the right and left insula, and right substantia nigra showed statistically significant higher reward-related response to dynamic ads as compared to static ads. The right ventral tegmental area and left substantia nigra showed significantly higher reward-related response to dynamic ads as compared to static ads before the FDR correction but not after. There was no significant interaction between food ad medium and child weight status. Adjusted models that includes a total screen time exposure as a covariate did not change the results in the 14 bilateral regions (Supplementary Table 1).

**TABLE 2:** Region-of-interest (ROI) analysis (*N* = 115).

		Unadjusted LME models^1,2,4^			Adjusted LME models^1,2,3,4^		
	*L/R*	*t*-value	*p-*value	FDR *q*-value	*t*-value	*p-*value	FDR *q*-value
Nucleus accumbens	R	−1.49	0.140	0.218	−1.49	0.138	0.215
	L	−1.20	0.232	0.325	−1.24	0.218	0.305
Orbitofrontal cortex	R	−0.94	0.351	0.406	−0.97	0.331	0.386
	L	−1.02	0.309	0.393	−1.07	0.287	0.365
Amygdala	R	5.34	**<0.001**	**<0.001**	5.34	**<0.001**	**<0.001**
	L	2.43	**0.016**	0.056	2.43	**0.016**	**0.048**
Insula	R	3.07	**0.003**	**0.019**	3.15	**0.002**	**0.014**
	L	2.31	**0.023**	0.064	2.42	**0.017**	**0.048**
Hypothalamus	R	−0.89	0.377	0.406	−0.89	0.373	0.402
	L	0.09	0.929	0.929	0.10	0.919	0.919
Ventral tegmental area	R	2.07	**0.039**	0.089	2.09	**0.037**	0.086
	L	1.95	0.052	0.091	1.94	0.054	0.094
Substantia nigra	R	2.94	**0.004**	**0.019**	2.94	**0.004**	**0.019**
	L	2.04	**0.044**	0.089	2.04	**0.043**	0.086

^1^Linear mixed effects models.

^2^FDR-corrected threshold at q < 0.05 was used.

^3^Covariates include BMI-z, age, sex, % caloric intake at preload, and physical activity.

^4^Bold values represent the statistical significance.

### Exploratory whole-brain analysis

**FIGURE 4:**
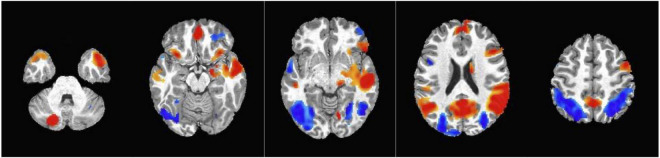
Clusters obtained from the whole brain analysis. Red = Activation in the positive direction (Dynamic > Static condition); Blue = Activation in the negative direction (Static > Dynamic condition).

Finding from the whole brain analysis are summarized in Table 3 and significant clusters are shown in Figure 4. Fourteen clusters showed a statistically significant higher response to dynamic ads compared to static ads. These included the left gyrus rectus, left middle temporal gyrus, left superior temporal gyrus, left superior frontal gyrus, right precuneus, right inferior frontal gyrus, right supplementary motor area, right cerebellum, and right and left calcarine fissure. Seven clusters showed a higher response to the static ads compared to dynamic ads including the left superior temporal gyrus, left inferior frontal gyrus, right cerebellum, right middle frontal gyrus, and left and right middle occipital gyrus. The results of the adjusted LME models were consistent with unadjusted models and all regions remained significant after FDR correction. Child weight status (healthy weight vs. having overweight and obesity) did not modify the effect of ad condition on neural response.

**TABLE 3:** Whole-brain analysis (*N* = 115)^1,2^.

Paired *t*-test (peak voxel)^3^						Adjusted LME models^4,5,6^	
Brain regions	Side	*X*	*Y*	*Z*	No. voxels^4^	*t*-value	FDR *q*-value
**Dynamic > Static condition**							
Middle temporal gyrus	L	60	−50	18	8,414	9.32	**<0.001**
	L	−52	−60	24	936	6.15	**<0.001**
Precuneus	R	2	−62	42	3,609	7.26	**<0.001**
	R	50	6	50	388	5.77	**<0.001**
Superior temporal gyrus: temporal pole	L	−36	10	−20	884	7.28	**<0.001**
Superior temporal gyrus	L	−54	−32	14	290	5.78	**<0.001**
Superior frontal gyrus, medial	L	2	62	18	725	5.99	**<0.001**
Inferior frontal gyrus, orbital part	R	52	24	−8	630	6.41	**<0.001**
Inferior frontal gyrus, triangular part	R	46	22	24	244	5.33	**<0.001**
Supplementary motor area	R	6	10	72	471	6.86	**<0.001**
Calcarine fissure and surrounding cortex	R	12	−82	6	391	6.04	**<0.001**
(Occipital lobe)	L	−8	−86	2	218	5.28	**<0.001**
Cerebellum crus^2^	R	−20	−74	−36	292	5.99	**<0.001**
Gyrus rectus	L	−2	48	−16	222	5.36	**<0.001**
**Static > Dynamic condition**							
Middle occipital gyrus	L	−32	−90	4	7,102	−7.37	**<0.001**
	R	30	−66	32	4,345	−6.79	**<0.001**
Superior temporal gyrus	L	−62	−12	4	1,025	−8.54	**<0.001**
Cerebellum crus^1^	R	32	−78	−20	550	−5.67	**<0.001**
Middle frontal gyrus, orbital part	R	34	48	−14	282	−5.26	**<0.001**
Inferior frontal gyrus, triangular part	L	−46	48	8	246	−4.60	**<0.001**
Inferior frontal gyrus, opercular part	L	−44	6	26	207	−4.55	**<0.001**

^1^Cluster size of *k* = 180.

^2^Each voxel has a voxel size of 2.5 × 2.5 × 2.5 mm.

^3^Reported MNI x, y, z coordinate is the peak voxel location within each cluster.

^4^Linear mixed effects models; Covariates include BMI-z, age, sex, % caloric intake at preload, and physical activity.

^5^Models based on the averaged activation in each cluster.

^6^Bold values represent the statistical significance.

## Discussion

This study examined whether advertising medium (dynamic or static) elicited a differential food-related neural responses in children. Children’s media use *via* TV, electronic mobile apps, and social media sites has increased dramatically between 2019 and 2021 (Rideout et al., 2022) and is likely increasing children’s exposure to unhealthy food advertising, which could motivate craving and lead to increased food consumption. Previous fMRI studies have compared the neural activation in response to food and non-food cues in either the dynamic medium (Gearhardt et al., 2014, 2020; Bruce et al., 2016; Rapuano et al., 2016, 2017; Masterson et al., 2019a) or static medium (Bruce et al., 2013, 2014; Fehse et al., 2017; Masterson et al., 2019b) of advertisement. As it is critical to examine children’s brain reactivity to food cues in a naturalistic and ecologically valid presentation, this is the first study that aimed to understand this gap in literature and compared the differential neural reward response to environmental food cues on two types of advertising mediums.

Several dopaminergic reward regions showed higher neural response in the dynamic medium of food ads compared to the static medium of food ads. These regions have been implicated in food cue responsivity previously using static food versus non-food images in the amygdala (Sayer et al., 2016; Melhorn et al., 2018; Luo et al., 2019; Roth et al., 2019) and insula (Schur et al., 2009; Scharmüller et al., 2012; Sayer et al., 2016; Roth et al., 2019), which are brain regions involved in salience, memory, and emotional regulation (Malik et al., 2011; Nummenmaa et al., 2012; Blechert et al., 2016; Roth et al., 2019). The ventral tegmental area and substantia nigra, areas characterized by their dopaminergic neurons and involved in dopamine transmission (Ikemoto, 2007; Kenny, 2011; Ilango et al., 2014), have also shown food cue responsivity to static images (Melhorn et al., 2018). Additionally, previous literature has found that the activation in the amygdala, insula, ventral tegmental area, and substantia nigra are related to food craving and appetitive motivation and regulation, (Pelchat et al., 2004; Siep et al., 2012; Dietrich et al., 2016; Douglass et al., 2017; Bond et al., 2020) which may further drive subsequent food consumption. Our findings suggest that children may be more responsive to food cues when presented in a dynamic medium, and further implies that dynamic ads may be more motivating and engaging to young children than static ads.

From the whole brain analysis, we found increased activation in the precuneus and the occipital lobe in response to the dynamic versus static ad condition, regions that are involved in identifying and detecting the salience of appetitive cue (Tang et al., 2012; Gearhardt et al., 2014; Rapuano et al., 2016). We also observed greater activation in the supplementary motor area to dynamic ads, and this may be indicative of activation of the action observation network where neurons in the motor cortex are active when observing another subject performing the action as if one is performing the action itself (Caspers et al., 2010).

Additionally, the whole brain analysis showed that the bilateral middle occipital gyrus and right middle frontal gyrus had greater activation to the static ad medium. The middle occipital gyrus is involved in visual processing and attention (Murdaugh et al., 2012), and middle frontal gyrus has been implicated in self-control (Harris A. et al., 2013) and response inhibition (Garavan et al., 1999). Studies that compared the neural response to the food versus non-food logos in younger children also found a food-cue-related response in the right occipital cortex (Bruce et al., 2014) and the right middle frontal gyrus (Bruce et al., 2013). Together, findings suggest that there may be activation in self-regulatory behavior networks when static food ads are present. Therefore static ads may play a distinct role in altering behavior. This may be an important consideration for policy makers as both static and dynamic ads are often displayed alongside one another, particularly on online platforms.

Though we did not examine the mechanisms by which dynamic ads elicit a greater reward response than static ads, we hypothesize that the narrative and emotional aspects of the dynamic food ads may increase their saliency compared to their static counterparts. Static ads such as banners on websites utilize powerful visuals and catch-phrases to increase brand exposure and awareness (Pieters and Wedel, 2004; Taylor et al., 2006; Edquist et al., 2011) while dynamic ads present messages using both visual images and audio recordings. A previous study that presented a content analysis of ads for children’s packaged foods and beverages showed that child-directed ads highlighted fun, taste, and humor (Emond et al., 2015). The narrative and emotional aspects of the dynamic food ads (versus static food ads) may draw more attention and prompt higher neural responses in younger children.

Collectively, the present findings have implications for the design and interpretation of future food-cue reactivity studies. Researchers should recognize that the medium of food cue presentation may impact the magnitude and location of observed brain response, and comparisons between studies should be cognizant of the food cue medium used. Future studies that assess food marketing exposure in children should distinguish between marketing *via* different mediums as they may have varied effects on eating behavior and other health-related outcomes.

Results from this study lend further support to the need for increased regulations regarding child-directed to food marketing. In the U.S., marketing regulations are voluntary but not statutory,^4^ which are consistently scrutinized as insufficient in reducing children’s exposure to marketing for unhealthy foods and drinks (Frazier and Harris, 2018; Jensen et al., 2022). The political will to support a government regulatory framework to reduce child-directed food marketing remains a challenge in the US, and more policy options to protect children from unhealthy TV food advertising are still needed (Harris J. L. et al., 2013; Boyland and Harris, 2017; Taillie et al., 2019; Fleming-Milici and Harris, 2020). Nevertheless, recent advances made by the Children’s Food and Beverage Advertising Initiative recognize the reach and power of online food marketing, and our findings suggest that the efforts to limit the ad medium may reduce the effectiveness of child-directed food marketing without enforcing more stringent restrictions on ad placement.

Our study has limitations that should be noted. First, because this study was embedded in a larger study, there was lack of randomization in the order of dynamic and static ad runs with the four dynamic runs always preceding the four static runs. We therefore cannot entirely rule out order effects. However, the runs and stimuli within those runs were sufficiently randomized. Additionally, the length of the fMRI scanning paradigm may have affected the scan quality given the younger age group. However, we had a fairly conservative motion screening threshold for excess motion, and no functional run was removed due to excess motion. Additionally, other studies have also successfully conducted fMRI paradigms that are similar or longer in length in similarly aged children (English et al., 2017; Adise et al., 2018; Masterson et al., 2019b). Second, our sample was largely white, non-Hispanic, and of a higher socioeconomic status, and therefore our results may not be generalizable to other populations. Future studies are needed to investigate whether these associations vary by ethnicity, race, and socioeconomic status. Third, the fMRI paradigm was conducted after the consumption of a full meal, which may have differentially reduced the neural response to food cues in the dynamic and static conditions (Goldstone et al., 2009). Future research should also explore concurrent presentation of static and dynamic food ads and or other outlets that combine presentation medium such as advergaming, a dynamic medium often accompanied by static advertising that is frequently used to market unhealthy foods and beverages.

## Conclusion

In conclusion, we show that the food advertising medium affects the neural reward response to food cues in children. Because greater neural activity in the regions involved in the dopaminergic pathways in response to food cues has been related to greater consumption (Lawrence et al., 2012; Masterson et al., 2019b; Gearhardt et al., 2020) and weight gain (Stice et al., 2010; Demos et al., 2012; Gearhardt et al., 2014), future research is needed to understand differential impacts of food advertising in different mediums on subsequent eating behaviors and unhealthy weight gain. Given the high current level of media exposure and constant exposure to food marketing across platforms, children are also constantly exposed to a variety of unhealthy food marketing, and it is crucial to understand the health effects of this new level of exposure.

## Data availability statement

The raw data supporting the conclusions of this article will be made available by the authors, without undue reservation.

## Ethics statement

The studies involving human participants were reviewed and approved by the Institutional Review Board at Dartmouth College. Written informed consent to participate in this study was provided by the participants’ legal guardian/next of kin.

## Author contributions

TM and DG-D designed and supervised the data collection and formulated the hypothesis. DY analyzed the data and wrote the first draft of the manuscript. All authors contributed to the various stages of the study, read and edited several draft versions, and approved the final manuscript.

## Footnotes

1
http://youtube.com/

2
http://instagram.com/

3
https://www.twitch.tv/

4
https://bbbprograms.org/programs/all-programs/cfbai
