# Corrigendum: Differential neural reward reactivity in response to food advertising medium in children

**DOI:** 10.3389/fnins.2023.1170370

**Published:** 2023-03-23

**Authors:** Dabin Yeum, Courtney A. Jimenez, Jennifer A. Emond, Meghan L. Meyer, Reina K. Lansigan, Delaina D. Carlson, Grace A. Ballarino, Diane Gilbert-Diamond, Travis D. Masterson

**Affiliations:** ^1^Department of Biomedical Data Science, Geisel School of Medicine at Dartmouth College, Lebanon, NH, United States; ^2^Department of Epidemiology, Geisel School of Medicine at Dartmouth College, Lebanon, NH, United States; ^3^Department of Psychological and Brain Science at Dartmouth College, Hanover, NH, United States; ^4^Department of Pediatrics, Geisel School of Medicine at Dartmouth College, Lebanon, NH, United States; ^5^Department of Psychology, Columbia University, New York, NY, United States; ^6^Department of Medicine, Geisel School of Medicine at Dartmouth College, Lebanon, NH, United States; ^7^Department of Nutritional Sciences, College of Health and Human Development, The Pennsylvania State University, University Park, PA, United States

**Keywords:** food cues, fMRI, neural reactivity, visual stimuli, children, static ad, dynamic ad

In the original article, there was an error in **Figure 3** as published. An error was caught with the ventral tegmental area (VTA) masks. We have identified the MNI coordinates for VTA, created a mask, and updated the analysis.

The corrected **Figure 3** and its caption appear below.

The corrected Figure 3 with caption:

**Figure 3:**
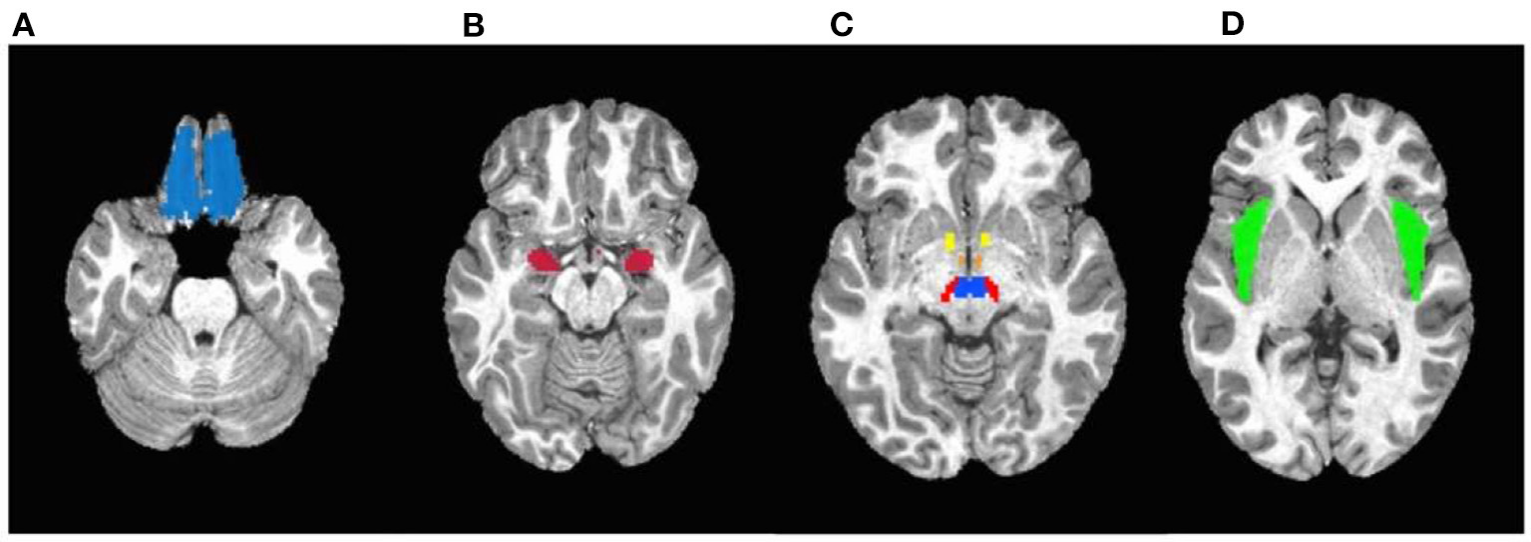
Masks used in the region-of-interest (ROI) analysis. **(A)** Orbitofrontal cortex. **(B)** Amygdala. **(C)** Yellow: Nucleus accumbens; Orange: Hypothalamus; Red: Substantia nigra; Blue: Ventral tegmental area. **(D)** Insula.

Following the incorrect mask used for VTA, there was an error in **Table 2**/**Supplementary Table 1** as published. Using the corrected mask, *t*-, *p*-, *q*-values for VTA changed. *q*-values (FDR-corrected statistical significance) for some other regions slightly changed because they are derived using the *p*-values of the multiple tests, but did not affect the interpretation of statistical significance. The VTA was statistically significantly associated with the dynamic advertising condition, but the statistical significance did not survive FDR correction.

The corrected **Table 2**/**Supplementary Table 1** appears below.

The corrected Table 2:

**Table 2:** Region-of-interest (ROI) analysis (*N* = 115).

		**Unadjusted LME models** ^**1,2,4**^			**Adjusted LME models** ^**1,2,3,4**^		
	***L/R***	***t*** **-value**	***p-*** **value**	**FDR** ***q*****-value**	***t*** **-value**	***p-*** **value**	**FDR** ***q*****-value**
Nucleus accumbens	R	−1.49	0.140	0.218	−1.49	0.138	0.215
	L	−1.20	0.232	0.325	−1.24	0.218	0.305
Orbitofrontal cortex	R	−0.94	0.351	0.406	−0.97	0.331	0.386
	L	−1.02	0.309	0.393	−1.07	0.287	0.365
Amygdala	R	5.34	**< 0.001**	**< 0.001**	5.34	**< 0.001**	**< 0.001**
	L	2.43	**0.016**	0.056	2.43	**0.016**	**0.048**
Insula	R	3.07	**0.003**	**0.019**	3.15	**0.002**	**0.014**
	L	2.31	**0.023**	0.064	2.42	**0.017**	**0.048**
Hypothalamus	R	−0.89	0.377	0.406	−0.89	0.373	0.402
	L	0.09	0.929	0.929	0.10	0.919	0.919
Ventral tegmental area	R	2.07	**0.039**	0.089	2.09	**0.037**	0.086
	L	1.95	0.052	0.091	1.94	0.054	0.094
Substantia nigra	R	2.94	**0.004**	**0.019**	2.94	**0.004**	**0.019**
	L	2.04	**0.044**	0.089	2.04	**0.043**	0.086

^1^Linear mixed effects models.

^2^FDR-corrected threshold at q < 0.05 was used.

^3^Covariates include BMI-z, age, sex, % caloric intake at preload, and physical activity.

^4^Bold values represent the statistical significance.

The corrected Supplementary Table 1:

**Supplementary Table 1:** Sensitivity analysis with total screen exposure time as a covariate.

		**Adjusted LME models** ^**1,2,3**^		
	***L/R***	***t*** **-value**	***p-*** **value**	**FDR** ***q*****-value**
Nucleus accumbens	R	−1.37	0.172	0.268
	L	−1.23	0.219	0.307
Orbitofrontal cortex	R	−0.87	0.384	0.419
	L	−1.04	0.300	0.382
Amygdala	R	5.26	**< 0.001**	**< 0.001**
	L	2.43	**0.016**	**0.045**
Insula	R	3.17	**0.002**	**0.014**
	L	2.43	**0.016**	**0.045**
Hypothalamus	R	−0.86	0.389	0.419
	L	0.13	0.895	0.895
Ventral tegmental area	R	2.09	**0.037**	0.086
	L	1.93	0.055	0.096
Substantia nigra	R	2.95	**0.004**	**0.019**
	L	2.00	**0.046**	0.092

Three corrections have been made to the main text due to the error in the VTA mask.

1. A correction has been made to **the abstract**, *Result*, line 46.

This sentence previously stated:

“From the ROI analyses, the right and left hemispheres of the amygdala and insula, and the right hemisphere of the ventral tegmental area and substantia nigra showed significantly higher responses for the dynamic food ad medium after controlling for covariates and a false discovery rate correction.”

The corrected sentence appears below:

“From the ROI analyses, the right and left hemispheres of the amygdala and insula, and the right hemisphere of the substantia nigra showed significantly higher responses for the dynamic food ad medium after controlling for covariates and a false discovery rate correction.”

2. A correction has been made to **the method**, *Region of Interest Analyses*, paragraph 1, line 312.

This sentence previously stated:

“Masks of these bilateral regions were generated using the Talairach Daemon and Montreal Neurological Institute (MNI) atlas using AFNI (Analysis of Functional NeuroImages version: 21.0.06, (Cox and Hyde, 1997) and are shown in Figure 3.”

The corrected sentence appears below:

“Masks of these bilateral regions were generated using the Talairach Daemon and Montreal Neurological Institute (MNI) atlas using AFNI [Analysis of Functional NeuroImages version: 21.0.06 (Cox and Hyde, 1997)]. The mask of the ventral tegmental area was defined by the sphere with a radius of 5 mm centered at MNI coordinate [4, −16, −10] (Carter, 2009). The ROI masks are shown in Figure 3.”

3. A correction has been made to **the results**, *ROI Analyses*, paragraph 1, line 357.

This sentence previously stated:

“Specifically, in both unadjusted and adjusted models and after the FDR correction, the right and left amygdala, the right and left insula, right ventral tegmental area, and right substantia nigra showed statistically significant higher reward-related response to dynamic ads as compared to static ads.”

The corrected sentence appears below:

“Specifically, in both unadjusted and adjusted models and after the FDR correction, the right and left amygdala, the right and left insula, and right substantia nigra showed statistically significant higher reward-related response to dynamic ads as compared to static ads. The right ventral tegmental area and left substantia nigra showed significantly higher reward-related response to dynamic ads as compared to static ads before the FDR correction but not after.”

The authors apologize for this error and state that this does not change the scientific conclusions of the article in any way. The original article has been updated.
