# Supplementary material for: Differential neural reward reactivity in response to food advertising medium in children
Source: Front Neurosci. 2023 Feb 1;17:1052384. doi: 10.3389/fnins.2023.1052384 (PMC9933514; doi:10.3389/fnins.2023.1052384)
Supplement: Supplementary file 1 [file Table_1.DOCX]

**Supplementary Table 1.** Sensitivity analysis with total screen exposure time as a covariate

|  | | **Adjusted LME Models^1,2,3^** | | |
| --- | --- | --- | --- | --- |
|  | *L/R* | *t*-value | *p-­*value | FDR *q*-value |
| Nucleus accumbens | R | -1.37 | 0.172 | 0.268 |
|  | L | -1.23 | 0.219 | 0.307 |
| Orbitofrontal cortex | R | -0.87 | 0.384 | 0.419 |
|  | L | -1.04 | 0.300 | 0.382 |
| Amygdala | R | 5.26 | **<0.001** | **<0.001** |
|  | L | 2.43 | **0.016** | **0.045** |
| Insula | R | 3.17 | **0.002** | **0.014** |
|  | L | 2.43 | **0.016** | **0.045** |
| Hypothalamus | R | -0.86 | 0.389 | 0.419 |
|  | L | 0.13 | 0.895 | 0.895 |
| Ventral Tegmental area | R | 2.09 | **0.037** | 0.086 |
|  | L | 1.93 | 0.055 | 0.096 |
| Substantia Nigra | R | 2.95 | **0.004** | **0.019** |
|  | L | 2.00 | **0.046** | 0.092 |

^1^Linear mixed effects models

^2^FDR-corrected threshold at q<0.05 was used

^3^Covariates include BMI-z, age, gender, % caloric intake at preload, physical activity, and total screen exposure time per week
