# Supplementary figures and images for: Differential neural reward reactivity in response to food advertising medium in children
Source: Front Neurosci. 2023 Feb 1;17:1052384. doi: 10.3389/fnins.2023.1052384 (PMC9933514; doi:10.3389/fnins.2023.1052384)

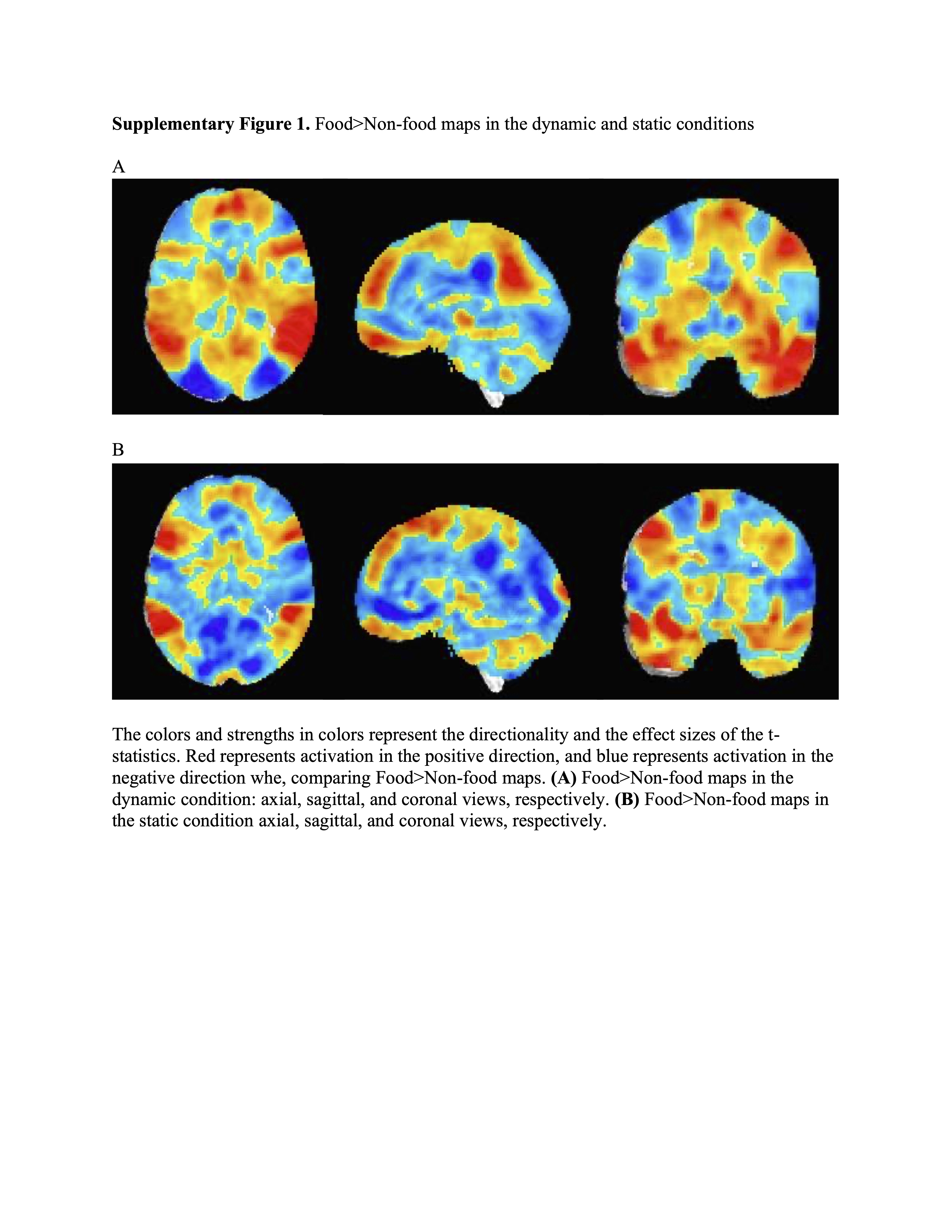

Supplement: Supplementary file 2 [file Image_1.jpg]
